# Can Environmentally-Specific Transformational Leadership Foster Employees’ Green Voice Behavior? A Moderated Mediation Model of Psychological Empowerment, Ecological Reflexivity, and Value Congruence

**DOI:** 10.3390/bs15070945

**Published:** 2025-07-12

**Authors:** Nianshu Yang, Jialin Gao, Po-Chien Chang

**Affiliations:** 1 School of Business, Macau University of Science and Technology, Taipa, Macau 999078, China; 3230003813@student.must.edu.mo (N.Y.); 3220004162@student.must.edu.mo (J.G.); 2 School of Management, Guangdong University of Science and Technology, Dongguan 523083, China

**Keywords:** green voice behavior, environmentally-specific transformational leadership, psychological empowerment, ecological reflexivity, person-supervisor value congruence

## Abstract

Employees’ green voice behavior (GVB), as a specific category of extra-role green behavior, plays a vital role in promoting a firm’s sustainable development. However, its underlying mechanism has not been sufficiently explored. Drawing on social learning theory (SLT), this study proposes a research model that examines the indirect influence of environmentally-specific transformational leadership (ESTFL) on GVB via psychological empowerment (PE) and ecological reflexivity (ER) as well as the moderating role of person-supervisor value congruence (PSVC). To achieve the research goals, we conducted a two-wave online survey via the convenience sampling method to collect data from 530 employees and 106 direct supervisors working in the manufacturing, hospitality and service, energy production, construction, transportation, information and communication, and finance industries in China. Regression analyses and CFA based on SPSS and Mplus were employed to test and validate the research model. Our findings show that PE and ER both partially mediated the positive association between ESTFL and GVB. Moreover, PSVC moderated the mediating effects of ESTFL on GVB via PE and ER. This study advances empirical research regarding how leadership impacts GVB by revealing dual cognitive mechanisms and identifying its boundary condition. It also offers managerial implications for leaders and enterprises in China to promote employees’ GVB and improve sustainable management.

## 1. Introduction

In recent decades, environmental issues have garnered worldwide attention (Ding et al., 2023; Holland & Albrecht, 2013; Robertson & Barling, 2013). As one of the largest manufacturing countries and carbon emitters, China has also placed a high priority on environmental issues (Wen & Liu, 2022). Undoubtedly, as major contributors to environmental problems, enterprises inevitably face pressure from shareholders, customers, governments, and other stakeholders (Daily et al., 2009; Ma & Ma, 2024). In order to address these environmental concerns, an increasing number of firms have begun adopting green management systems, policies, and practices (F. Liu & Qi, 2022; Renwick et al., 2013; J. Zhang et al., 2021). However, due to the complexity of environmental issues (Iqbal et al., 2023), merely relying on these formal organizational interventions from the top is insufficient (Morrison & Milliken, 2000). Actually, most greening efforts rely on employees’ voluntary engagement beyond their job duties (Kim et al., 2017; F. Liu & Qi, 2022). Thus, it is crucial for firms to stimulate employees to participate in green affairs actively and voluntarily, especially for SMEs that often lack enough resources to deal with such challenges compared with large firms (Zaid & Yaqub, 2024).

Currently, researchers are mainly focused on employees’ organizational citizenship behavior toward the environment (OCBE) or other extra-role behaviors (Luu, 2024; Murillo-Ramos et al., 2024), with little knowledge about green voice behavior (GVB) (Y. Cheng et al., 2022; S. Hu et al., 2025; Nazeer et al., 2025). GVB refers to a proactive extra-role behavior conveying ideas and suggestions related to the environment in the workplace even when others disagree (Aboramadan et al., 2021; Ari et al., 2020; J. Zhang et al., 2024). It plays a critical role in avoiding potential environmental problems, providing better eco-friendly managerial decisions, and promoting organizations’ sustainable development (Morrison, 2011; Murillo-Ramos et al., 2024). However, employees are often reluctant to communicate their opinions and concerns to others even though they know their suggestions benefit their organizations (Morrison, 2011) because voice is a risky and costly behavior that may lead to the employees being perceived as troublemakers or opponents as it usually challenges the status quo or embarrasses leaders (Chou & Barron, 2016; Murillo-Ramos et al., 2024; Nazeer et al., 2025). Not surprisingly, finding factors that stimulate employees to express green-related ideas freely has become one of the most important topics (W. Liu et al., 2010; Nazeer et al., 2025).

The present study addresses this gap by investigating the mechanisms behind employee GVB. Previous studies regarding the antecedents of GVB mainly focused on green HRM (Z. Liu et al., 2024, 2025; Murillo-Ramos et al., 2024), corporate social responsibility (Afsar et al., 2020; Crucke et al., 2022; Shah et al., 2021), and green mindfulness (Kim et al., 2017; Murillo-Ramos et al., 2024), which explored this topic from the perspectives of organizations and employees. However, research focusing on how leadership promotes employees’ GVB is far from sufficient (Aboramadan et al., 2023; Crucke et al., 2022). It has been pointed out that leadership is prominent in affecting and fostering the attitudes, values, and behaviors of employees (Alwheshi et al., 2024; Mughal et al., 2022; Robertson & Barling, 2013), especially in China with a high-power distance culture. Moreover, extensive studies have verified the significant effect of leadership on voice behavior (Detert & Burris, 2007; Ilyas et al., 2021; C. Liu et al., 2023; W. Liu et al., 2010). Accordingly, responding to calls for further studies on this issue (Afsar et al., 2018; Crucke et al., 2022; Ilyas et al., 2021), we employed social learning theory (SLT) (Bandura, 1977) to examine the impact of direct leaders on employees’ GVB considering their significant and direct influence on employees due to frequent interactions (Crucke et al., 2022; Robertson & Barling, 2013). Given the complexity and potential challenges in addressing environmental issues, firms usually require leaders who are inspirational, motivated and transformation-focused. Thus, this study particularly discusses the effect of environmentally-specific transformational leadership (ESTFL) on GVB. ESTFL is regarded as a form of transformational leadership that focuses on influencing employees’ and organizations’ pro-environmental initiatives through idealized influence, inspirational motivation, intellectual stimulation, and individualized consideration (Chen & Chang, 2013; Robertson & Barling, 2013). This is a key leadership style in fostering green behaviors (Crucke et al., 2022) as it conveys the values of sustainability to subordinates and encourages them to take eco-friendly actions (Ding et al., 2023; Ramus & Steger, 2000; Robertson & Barling, 2013).

Additionally, to further reveal the “black box” of how ESTFL influences employee GVB, we propose that psychological empowerment (PE) and ecological reflexivity (ER) are the dual mechanisms that explain this relationship based on SLT. PE is defined as an intrinsic motivation containing meaning, impact, self-determination, and competence (Ilyas et al., 2021; Spreitzer, 1995), while ER refers to the extent to which employees consider whether their task goals and procedures align with green development and adapt them to green development paradigms (J. Liu et al., 2024; Wang et al., 2021). In line with SLT, it is reasonable to propose that ESTFL exerts an influence on the employees’ PE (Raub & Robert, 2013; Spreitzer, 1995) and ER, which in turn stimulates them to make green-related suggestions and comments in the workplace. Specifically, when ESTFL leaders demonstrate their commitment to green issues and appreciation for green actions, their followers are more likely to regard green management as meaningful and demonstrate greater confidence and initiative in expressing their ideas and suggestions on environmental matters. Meanwhile, through reflecting on ecological issues, employees recognize that they can make constructive suggestions to solve environmental problems and enhance green management. As a result, they are more motivated to conduct GVB.

We also test the model of ESTFL and GVB through identifying a crucial boundary condition. SLT proposes that the similarity of leaders and subordinates can significantly impact the learning process (Li & Xu, 2019). Thus, we further examine the boundary role of person-supervisor value congruence (PSVC), which reflects the level of value consistency between employees and leaders (Astakhova, 2016; Jensen et al., 2019; Yue et al., 2023). It has been regarded as a key contextual moderator in the literature on leadership (K. Cheng et al., 2019; He et al., 2024). Specifically, we theorize that PSVC moderates the indirect effect of ESTFL on GVB via PE and ER. When PSVC is high, the influences of ESTFL on GVB through PE and ER can be stronger, and vice versa.

This study aims to fill research gaps in the literature regarding the influence of leadership on GVB. By examining ESTFL exhibited by direct leaders in the workplace, we conceptualize ESTFL as establishment and reinforcement processes of employees’ PE and ER, providing new insights into the effectiveness of promoting employees’ GVB through ESTFL. In this sense, we also advance ESTFL research by identifying cognitive paths through which leadership fosters the employees’ GVB. In addition, we aim to draw a fuller picture of SLT and expand its application. Currently, few studies have focused on outcome expectancy (Lian et al., 2012) or two expectations (Wang et al., 2018). Moreover, to date, no definitive consensus has been reached regarding the relationship between efficacy and outcome expectancy (Li & Xu, 2019; Williams, 2010). This study will illuminate two crucial cognitive mechanisms of the social learning process by exploring the dual mechanisms of efficacy expectation (i.e., PE) and outcome expectation (i.e., ER) in the green management context. Taken together, using SLT as a theoretical framework, our research will further explain why and how ESTFL influences employees’ GVB.

To achieve these research objectives, this study is structured as follows. Section 2 establishes the theoretical framework of the study. Section 3 describes the research methodology including the data collection procedures. Section 4 presents the analytical techniques and empirical findings of the study. Finally, in Section 5, we provide an in-depth discussion of the theoretical and managerial implications as well as the limitations and future research directions.

## 2. Theoretical Foundation and Research Hypotheses

### 2.1. Underlying Theory

Bandura (1977) developed SLT. This theory posits that individuals learn social behaviors through observing and imitating others (Bandura, 1977). Moreover, the learning process can be realized through two intermediary cognitive processes including efficacy expectation and outcome expectation. Efficacy expectation refers to the extent to which employees believe they can execute certain behaviors, while outcome expectation represents employees’ estimation of the likelihood of outcomes resulting from behaviors (Li & Xu, 2019; Wang et al., 2018). When individuals’ efficacy and outcome expectations are improved, their learning efforts will be strengthened. Furthermore, SLT suggests that the effectiveness of social learning can be affected by contextual factors (Li & Xu, 2019).

This research is grounded in SLT, which provides a robust theoretical framework for explaining GVB in organizational contexts. Especially in Chinese culture, leaders often play crucial roles in affecting employee attitudes and behaviors. In addition, PE and ER, as efficacy expectation and outcome expectation, respectively, can efficiently explain the relationship between ESTFL and GVB. Furthermore, the value similarity between leaders and followers can play a boundary role in this model.

### 2.2. Environmentally-Specific Transformational Leadership and Green Voice Behavior

The concept of voice originates from the idea that employees identify some problems or opportunities to enhance personal and organizational welfare (Detert & Burris, 2007; Hirschman, 1970). Just as described by Van Dyne and LePine (1998), it is a type of voluntary behavior that is both promotive and challenging. Being promotive means that voice can be regarded as a proactive behavior that is future-oriented and constructive in intent, while being challenging implies that it is change-oriented and may harm relationships (Morrison, 2011; Raub & Robert, 2013; Van Dyne & LePine, 1998). Based on these, GVB is viewed as a voluntary and eco-friendly behavior that entails making novel pro-environmental suggestions and advocating for changes to existing protocols even when others disagree (Aboramadan et al., 2021; Murillo-Ramos et al., 2024). Implicit in the definition is that GVB may lead to “relatedness losses” and “existence losses” (Detert & Burris, 2007) due to its challenge to the status quo or leaders and colleagues (Morrison, 2011; Raub & Robert, 2013). Undoubtedly, employees require sufficient motivation to perform such behaviors.

Extensive research has discussed the influence of leaders on voice behaviors (Crucke et al., 2022; Detert & Burris, 2007; C. Liu et al., 2023). On the one hand, leaders possess the power and resources to implement modifications. On the other hand, they have the power to reward or punish subordinates. Thus, the leaders’ acceptance of changes is a crucial determinant of the employees’ willingness to voice (Detert & Burris, 2007). ESTFL, as a type of transformational leadership focusing on pro-environmental initiatives (Robertson, 2018; Robertson & Barling, 2013), is characterized by green inspirational motivation, idealized influence, individualized consideration, and intellectual stimulation (Bass, 1999; Bass & Avolio, 1991; Chen & Chang, 2013; X. Liu & Yu, 2023; Robertson & Barling, 2013). It attaches great importance to organizational long-term environmental objectives and highlights the establishment of shared green values and visions (Robertson & Carleton, 2018). Moreover, ESTFL leaders tend to encourage their subordinates to think and behave creatively and in an eco-friendly manner (Bass, 1990; Bass & Avolio, 1991). Thus, it is an effective leadership style in promoting employee GVB (Crucke et al., 2022).

In accordance with SLT (Bandura, 1977; Li & Xu, 2019), we argue that ESTFL leaders set examples for employees to learn and imitate (Bass, 1999), thereby enhancing employee positive voice behavior (Aboramadan et al., 2023; Crucke et al., 2022). Specifically, leaders high in environmental idealized influence will be regarded as environmental role models by their subordinates. In this case, followers tend to establish green values that are consistent with their leaders and propose eco-friendly suggestions for sustainable development (Ma & Ma, 2024; Simola et al., 2010). Furthermore, individualized considerate leaders usually pay much attention to their employees’ difficulties in addressing green-related affairs and to their contributions to green issues (Robertson & Barling, 2013). Consequently, the followers will trust their leaders and interact with them more effectively, which helps them overcome their worries and fears in voicing their green ideas (Ilyas et al., 2021). Likewise, supervisors with high intellectual stimulation usually encourage their followers to cope with green issues in creative ways (Boiral et al., 2015; Robertson & Barling, 2013), which means that leaders allow challenges to the status quo and appreciate novel ideas (Avolio et al., 2004; W. Liu et al., 2010). Thus, employees feel more competent and willing to perform voice behavior. Furthermore, leaders who exhibit green inspirational motivation often set high environmental goals and inspire their employees to strive for these goals. Therefore, to better meet the leaders’ expectations, the employees’ observational learning intentions will be strengthened, making them more powerful in performing GVB. In conclusion, ESTFL leaders can act as strong role models by fostering strong relational bonds with their followers (Burns, 1978; A. N. Khan & Khan, 2022), and also building an open, inclusive, and inspiring climate that effectively facilitates the employees’ learning processes, thereby improving their GVB (Mukhtar et al., 2025). Accordingly, this research proposes:

**Hypothesis** **1** **(H1).** *Environmentally-specific transformational leadership (ESTFL) is positively associated with green voice behavior (GVB)*.

### 2.3. The Mediating Role of Psychological Empowerment

PE is regarded as a psychological state shaped by the work environment (Spreitzer, 1995; X. Zhang & Bartol, 2010). Conger and Kanungo (1988) viewed it as a sense of self-efficacy, while Thomas and Velthouse (1990) defined it as an increased intrinsic motivation encompassing impact, competence, meaningfulness, and choice. Based on these, Spreitzer (1995) further defined PE as a motivational concept containing meaning, impact, self-determination, and competence (Ilyas et al., 2021; Raub & Robert, 2010; Seibert et al., 2011; Spreitzer, 1995). Specifically, meaning reflects the employees’ sense of importance and value of their jobs and goals (Spreitzer, 1995; Thomas & Velthouse, 1990). Impact indicates that employees believe that they can impact the organizations’ work outcomes (Spreitzer, 1995, 1996). Self-determination is a feeling of autonomy in deciding how to complete jobs (Raub & Robert, 2013; X. Zhang & Bartol, 2010). Finally, competence refers to an individual’s belief in his or her ability to successfully perform tasks (Spreitzer et al., 1997).

SLT indicates that the learning process is not only behavioral but also a psychological process (Guo et al., 2021). This means that leadership influences followers by changing their cognitions including efficacy expectancy and outcome expectancy (Li & Xu, 2019). According to the definition of PE, it is also a form of efficacy expectancy. Some researchers have verified that PE plays a mediating role in the relationship between leadership and employee voice behavior (Y. Hu et al., 2018; Hwang et al., 2023; Ilyas et al., 2021). Therefore, we suggest that PE can also mediate the relationship between ESTFL and GVB (Rietze & Schölmerich, 2025). In other words, ESTFL leaders can improve their followers’ PE, thereby stimulating them to conduct GVB.

A meta-analysis research pointed out that leadership is one of the most crucial factors influencing PE (Schermuly et al., 2022; Seibert et al., 2011). Among the leadership styles, ESTFL also significantly impacts the employees’ PE (Priyadarshini et al., 2023). First, ESTFL offers the necessary green-related information and resources, such as green visions, regulations, and policies as well as funds and green skills, which can help followers understand the organizations’ green goals deeply and improve their capabilities to implement green measures. As a result, it enhances the followers’ sense of meaning and competence (Gurmani et al., 2021; Spreitzer, 1996). Second, ESTFL leaders often encourage employees to actively participate in green-related decision-making. Moreover, they tend to recognize and express their appreciation for their followers’ contributions to green management, which makes employees perceive their importance to organizations (Spreitzer, 1996), thus enhancing their sense of impact. Third, ESTFL leaders can also foster their employees’ feelings of autonomy, as they usually encourage followers to think and deal with environmental problems creatively (Priyadarshini et al., 2023). In conclusion, as a motivational role model, ESTFL can enhance the employees’ sense of empowerment (Schermuly et al., 2022).

Additionally, due to the discretionary and risky nature of GVB, employees may be reluctant to engage in GVB (C. Liu et al., 2023), which offers theoretical and empirical validity for the association between PE and GVB (Raub & Robert, 2013). Thus, we suggest that psychologically empowered employees usually feel more motivated to engage in voice behaviors (Rietze & Schölmerich, 2025). More specifically, employees high in PE often exhibit greater confidence in their suggestion-making capabilities. They believe in their importance to their organizations’ environmental outcomes and feel more autonomous in making suggestions and performing jobs, which makes them more willing to take risks (Jeung & Yoon, 2018). In other words, a high level of PE can provide a high efficacy expectancy for employees to perform GVB. On the contrary, less empowered employees are discouraged from expressing their eco-friendly ideas freely because they think it will challenge the status quo and lead to negative results.

Based on the above discussions, this study suggests that ESTFL leaders can satisfy the self-efficacy expectancy of their employees through PE, which in turn stimulates the employees’ GVB. This aligns with the role of efficacy expectancy in SLT. Therefore, we propose:

**Hypothesis** **2** **(H2).** *Psychological empowerment (PE) mediates the relationship between environmentally-specific transformational leadership (ESTFL) and green voice behavior (GVB)*.

### 2.4. The Mediating Role of Ecological Reflexivity

ER, as a cognitive process, is a relatively new concept. Based on the definition of team reflection (Schippers et al., 2007), J. Liu et al. (2024) defined ER as the extent to which employees deliberate on whether their task goals and procedures align with the requirements of sustainable development and adapt them to ecological development paradigms. It involves two major dimensions, reflecting upon past green-related work and planning for future actions (De Jong & Elfring, 2010). To date, it has been less empirically explored. However, ER is critical in addressing environmental problems (Enninga & Yonk, 2023). Thus, we need to explore its mechanisms further. Previous studies have shown that leadership can affect employee behaviors and performance through influencing their level of reflection (He et al., 2024; Schippers et al., 2008). Therefore, ER may potentially act as a mediator between ESTFL and GVC.

Based on SLT, outcome expectancy is another intermediate cognition influencing the learning process (Wang et al., 2018). The encouragement of ESTFL leaders for creative green ideas and actions can intensify their employees’ social learning willingness, improving their ER levels. As a result, they can quickly identify problems and formulate solutions, which strengthens their expectancy for positive outcomes, thereby stimulating their voice behaviors. This means that leaders can promote their employees’ GVB through changing their perception of the outcomes.

Specifically, SLT suggests that employees tend to observe and imitate leaders who are charismatic and credible (Brown et al., 2005; Zheng et al., 2020). Undoubtedly, ESTFL leaders, as typically considerate and inspiring role models, will significantly affect their followers’ cognition and behaviors. When followers perceive their leaders attach great importance to sustainability, encourage active engagement in green affairs, and solve environmental issues in novel methods, they tend to reflect upon ecological issues and deal with green-related problems positively and actively.

In addition, ER is a key virtue of dealing with environmental problems effectively (Enninga & Yonk, 2023; Pickering, 2019). Prior studies have indicated that it can enhance employees’ creative thinking and expectations for the future, thus enabling them to better learn from their leaders and improve their green behaviors (J. Liu et al., 2024). On the one hand, ER stimulates employees to consider environmental issues creatively in their jobs (LePine et al., 2008), thereby making them more capable of finding environmental problems. On the other hand, ER helps employees to exchange and integrate various information and resources in the workplace (Monks et al., 2016), which makes them propose effective solutions. When they really perceive that they can point out problems and offer solutions to help deal with previous problems, avoid potential problems, and improve green performance through GVB, their belief that GVB is useful will be strengthened. Thus, they are more likely to perform this behavior, as encouraged by their leaders (J. Liu et al., 2024).

In summary, based on SLT, the outcome expectation of followers who are exposed to an ESTFL leader can be met through ER, which makes them more willing to voice their opinions and suggestions on green-related matters. This aligns with the role of outcome expectancy in SLT, which provides a new perspective for better understanding the relationship between ESTFL and GVB. In accordance with these discussions, we suggest:

**Hypothesis** **3** **(H3).** *Ecological reflexivity (ER) mediates the relationship between environmentally-specific transformational leadership (ESTFL) and green voice behavior (GVB)*.

### 2.5. The Moderating Roles of Person-Supervisor Value Congruence

SLT indicates that social learning does not happen in a vacuum. The similarity of leaders and followers can significantly influence the effectiveness of social learning (Li & Xu, 2019). He et al. (2024) posited that values are fundamental principles that shape the attitudes and behaviors of employees, so PSVC, which refers to the alignment of employee values and their leaders (Astakhova, 2016; Hoffman et al., 2011; Jensen et al., 2019), needs to be systematically addressed in the leadership context (Erdogan et al., 2004; Lee et al., 2017). This alignment can foster employee imitation of the leaders (Lan et al., 2020; Yue et al., 2023). When employees have high value congruence with their supervisors, they pay more attention to their leaders’ values and behaviors and are more inclined to follow their leaders’ requirements and emulate their leaders’ behaviors (Lau et al., 2007).

Previous research has verified that PSVC can moderate the relationship between leaders and employees (Akhtar et al., 2022; K. Cheng et al., 2019; Peng et al., 2023; Yao et al., 2024; Yue et al., 2023), which means that employees may respond differently, even when they work with the same leaders (Yue et al., 2023). Therefore, we suggest that PSVC also moderates the influence of ESTFL on PE as well as on ER. Specifically, when employees have high value congruence with their ESTFL leaders, they will exhibit higher levels of PE and ER. On the one hand, congruent values can enhance the employees’ precise and in-depth comprehension of their leaders’ green visions and goals (He et al., 2024), thereby increasing the employees’ sense of self-efficacy as well as facilitating their better reflection on environmental matters. On the other hand, when followers recognize that they have similar values and beliefs on green issues with their leaders, they can better adapt to changes in green management, thus completing green-related tasks more quickly. As a result, they will be more confident in participating in green management, and will also propose more constructive suggestions. In addition, employees who are consistent with their leaders are more likely to treat their leaders as trustworthy and admirable role models (K. Cheng et al., 2019) and then actively internalize their leaders’ green values and behaviors, which can deepen the employees’ PE and ER.

In contrast, low PSVC may obstruct the influence of leaders on followers (Z. Liu et al., 2023). On the one hand, employees who have low value congruence with leaders fail to fully understand the reasons and intentions behind these behaviors (He et al., 2024). On the other hand, they may view the leaders’ green values and actions as meaningless and valueless. Consequently, it can reduce the followers’ sense of self-efficacy and willingness to consider ecological issues. Especially when value incongruence exists between employees and leaders, employees may experience dissatisfaction or incompetence in addressing green-related challenges. Undoubtedly, this can significantly lower the employees’ sense of PE and hinder their ER.

In conclusion, SLT implies that the relationship between ESTFL and the followers’ PE and ER varies as a function of PSVC. To summarize, we propose:

**Hypothesis** **4a** **(H4a).** *Person-supervisor value congruence (PSVC) moderates the relationship between environmentally-specific transformational leadership (ESTFL) and psychological empowerment (PE), such that this relationship is stronger when person-supervisor value congruence (PSVC) is higher*.

**Hypothesis** **4b** **(H4b).** *Person-supervisor value congruence (PSVC) moderates the relationship between environmentally-specific transformational leadership (ESTFL) and ecological reflexivity (ER), such that this relationship is stronger when person-supervisor value congruence (PSVC) is higher*.

Drawing on the hypotheses that PSVC moderates the effects of ESTFL on PE and ER, and considering that the effect of ESTFL on GVB is mediated through PE and ER, it is logical to further propose that PSVC moderates the mediating mechanism of PE between ESTFL and GVB as well as ER between ESTFL and GVB. Specifically, when employees feel more congruent with their supervisors, they tend to foster high PE and ER through learning from and imitating their ESTFL leaders. As a result, they will feel more confident and meaningful in expressing their green-related opinions and suggestions in the workplace. On the contrary, when the PSVC is low, the leaders’ positive modeling role in followers may be weakened. In this case, the employees’ PE and ER may be undermined, failing to adequately fulfill the followers’ efficacy and outcome expectations, which ultimately reduces voice behaviors. Consequently, we propose:

**Hypothesis** **5a** **(H5a).** *Person-supervisor value congruence (PSVC) moderates the indirect effect of environmentally-specific transformational leadership (ESTFL) on green voice behavior (GVB) through psychological empowerment (PE), such that the indirect effect is stronger when person-supervisor value congruence (PSVC) is higher*.

**Hypothesis** **5b** **(H5b).** *Person-supervisor value congruence (PSVC) moderates the indirect effect of environmentally-specific transformational leadership (ESTFL) on green voice behavior (GVB) through ecological reflexivity (ER), such that the indirect effect is stronger when person-supervisor value congruence (PSVC) is higher*.

**Figure 1:**
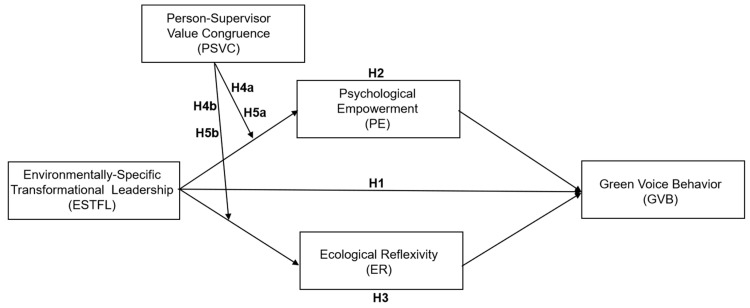
Theoretical framework.

In conclusion, this research proposes the following theoretical framework (see Figure 1).

## 3. Research Method

### 3.1. Procedures

This study employed the convenience sampling method and collected data with online questionnaires. To ensure that the samples aligned with the research context, we collected data from SMEs in China that have implemented environmental management systems or green-related initiatives, guaranteeing that the participants were indeed working in contexts requiring positive engagement in green management. Furthermore, to ensure that the participants had a better understanding of the organizations’ environmental policies and leadership styles, this study selected employees and supervisors who had worked in their firms or departments for at least one year, thereby enhancing the authenticity and validity of the data. To minimize common method bias, we used the time-lagged survey method to gather data in two waves from followers and their supervisors (Podsakoff et al., 2003).

At the beginning of the two-wave surveys, we explained the academic purpose and confidentiality principle of the study to all employees and supervisors to ensure their voluntary participation. Finally, a total of 800 employees from Chinese SMEs covering the manufacturing, hospitality and service, energy production, construction, transportation, information and communication, and finance industries participated in our study. During the first phase (Time 1), we invited employees to rate ESTFL and PSVC as well as provide demographic information. After four weeks (Time 2), the same employees who had completed the first wave were asked to complete PE and ER questionnaires. Meanwhile, we invited their direct supervisors to evaluate their GVB. To facilitate matching the questionnaires later, the employees were required to provide the last four digits of their phone number, and supervisors were also asked to fill in the last four digits of their followers.

### 3.2. Participants

In Time 1, we provided questionnaires to the employees and received 630 valid responses with an effective response rate of 78.8%. Four weeks later, questionnaires were distributed to 630 employees who had finished the first stage, and questionnaires were distributed to 114 direct supervisors. Finally, after removing the missing and invalid questionnaires, 530 valid dyadic data with an effective response rate of 84.1% were obtained.

According to the descriptive analysis of the participants in Table 1, 60.4% of the employees were male, while 39.6% were female. In addition, employees aged below 30, between 30 and 40, and above 40 accounted for 32.8%, 63.0%, and 4.2%, respectively. Regarding education, 1.9% held high school degrees or below, 6.8% held junior college degrees, 88.7% held bachelor’s degrees, and 2.6% possessed master’s degrees or above. For work tenure, 66.4% of employees had worked for 1 to 5 years, 32.6% for 5 to 10 years, and 1.0% over 10 years. In the supervisor sample, 68.9% were male. Regarding their ages, 49.1% were between 30 and 40, while 50.9% were above 40. They all held bachelor’s or master’s degrees. In addition, 82.1% of supervisors had been employed in their firms for over 5 years.

### 3.3. Measures

To ensure the quality and equivalence of the questionnaire, all scales were from existing ones that had been developed and verified. Due to all scales being from English journals, we invited bilingual foreign-language experts to adapt and translate them into Chinese with the back-translation procedure (Brislin, 1970, 1980). Participants rated each of the items using a 5-point Likert-type scale option from “1” (strongly disagree) to “5” (strongly agree).

#### 3.3.1. Environmentally-Specific Transformational Leadership

ESTFL was rated with 12 items introduced by Robertson (2018). This contains four dimensions: environmental idealized influence, individualized consideration, inspirational motivation, and intellectual stimulation. A sample item was “My supervisor motivates me to work in an environmentally friendly manner”. The Cronbach’s alpha coefficient was 0.75.

#### 3.3.2. Green Voice Behavior

We employed three items adapted from Aboramadan et al. (2021), Van Dyne and LePine (1998) to measure the GVB. This variable was rated by supervisors to avoid common method bias due to the self-reports. A sample item was “(S)he makes recommendations concerning environmental issues which affects her/his work”. The Cronbach’s alpha was 0.76.

#### 3.3.3. Psychological Empowerment

PE was measured with a 12-item scale developed and validated by Spreitzer (1995). It has four dimensions including meaning, competence, self-determination, and impact. A sample item was “The work I do is very important to me”. The Cronbach’s alpha was 0.76.

#### 3.3.4. Person-Supervisor Value Congruence

PSVC was measured by three items applied and validated by Hoffman et al. (2011). A sample item was “My supervisor’s values provide a good fit with the things I value”. The Cronbach’s alpha was 0.76.

#### 3.3.5. Ecological Reflexivity

We used four items from J. Liu et al. (2024) adapted from Schippers et al. (2013) and De Dreu (2007) to measure the ER. A sample item was “I often review my past working methods with environmental protection as the goal”. The Cronbach’s alpha for this scale was 0.77.

#### 3.3.6. Control Variables

Prior research has shown that demographic variables can influence employees’ green behaviors (M. A. S. Khan et al., 2019), so the study viewed the employees’ gender, age, education, and experience as control variables in the process of examining the research model. Specifically, gender was dummy-coded (“0” = male, “1” = female). Education had four options (“1” = high school or below; “2” = junior college; “3” = bachelor; “4” = master or above). Additionally, the number of tenure working in existing departments or organizations served as the indicator of experience.

## 4. Data Analysis and Results

In this study, we used SPSS 21.0 and Mplus 8.3 to perform common method bias (CMB), descriptive statistics, correlation analysis, and confirmatory factor analysis (CFA). Additionally, to examine the moderated mediation model, we followed Preacher et al.’s (2007) method by adopting Hayes’ PROCESS macro for SPSS (Hayes, 2013).

### 4.1. Common Method Bias

In this study, ESTFL, PE, ER, and PSVC were all self-rated by the employees. Although we conducted multiple procedural methods to mitigate CMB, we could not entirely avoid its potential influence. Therefore, we employed Harman’s single-factor test to examine the CMB (Podsakoff et al., 2003). Results showed that the first factor explained 20.77% of the total variance, below the threshold of 50%, suggesting no significant CMB issues in this study. In addition, we also used the unmeasured latent variable technique (UMLV) and full collinearity analysis to test CMB. In Table 2, the UMLV test results showed no substantial evidence of common method bias (ΔCFI = +0.01 < 0.1; ΔTLI = +0.02 < 0.1; ΔRMSEA = 0.00 < 0.05). The variance inflation factors (VIFs) of ESTFL (1.14), PE (1.47), and ER (1.34) were all less than the threshold of 3.3, indicating no multicollinearity concerns. Thus, CMB is unlikely to significantly affect the results.

### 4.2. Confirmatory Factor Analysis

We adopted confirmatory factor analysis (CFA) to examine the convergent and discriminant validity. In Table 2, the five-factor model, including ESTFL, PE, ER, PSVC, and GVB, fitted the data well [χ^2^(125) = 252.76, CFI = 0.96, TLI = 0.95, SRMR = 0.03, and RMSEA = 0.04], which satisfied the standard criteria of CFA (χ^2^/df < 3, CFI > 0.90, TLI > 0.90, SRMS < 0.08 and RMSEA < 0.08) (Kyndt & Onghena, 2014).

In this study, we analyzed the average variance extracted (AVE) and composite reliability (CR) values. According to Table 3, the results showed that the CR values for all variables exceeded the standard threshold of 0.7, indicating strong internal consistencies. Meanwhile, the AVE values of ER, PSVC, and GVB all met the standard threshold of 0.5, while the AVE values of ESTFL and PE fell within acceptable ranges (Chin, 1998). In addition, the square root values of AVE for ESTFL, PE, ER, PSVC, and GVB were all higher than their correlation coefficients with other variables. Consequently, the convergent validity and discriminant validity of all variables were sufficient for further study.

### 4.3. Descriptive Statistics and Correlations

Table 3 presents the means, standard deviations, and correlations. These indicated that ESTFL positively impacts PE (r = 0.35, *p* < 0.01), ER (r = 0.19, *p* < 0.01), and GVB (r = 0.39, *p* < 0.01). Additionally, both PE (r = 0.49, *p* < 0.01) and ER (r = 0.61, *p* < 0.01) showed positive and significant associations with GVB. These findings offer preliminary support for Hypothesis 1.

### 4.4. Hypotheses Testing

In Table 4, the results showed that ESTFL was positively associated with GVB (B = 0.39, *p* < 0.001), as predicted in Hypothesis 1. Additionally, we proposed positive relationships between ESTFL and GVB through PE as well as ER. According to MacKinnon et al. (2012), a direct relationship does not always imply a mediation effect. Therefore, to examine the significance of the mediation effects, we applied the bootstrapping technique. In line with standard practices, mediation is deemed significant when the 95% confidence interval, based on 5000 resamples, excludes zero (Hayes, 2013; Preacher & Hayes, 2008). The bootstrapping findings revealed the positive effect of ESTFL on GVB through PE (95%CI [0.04, 0.16]), implying partial mediation. Similarly, the effect of ESTFL on GVB via ER was also significant (95%CI [0.04, 0.29]), signifying partial mediation. Thus, Hypotheses 2 and 3 are supported.

**Figure 2:**
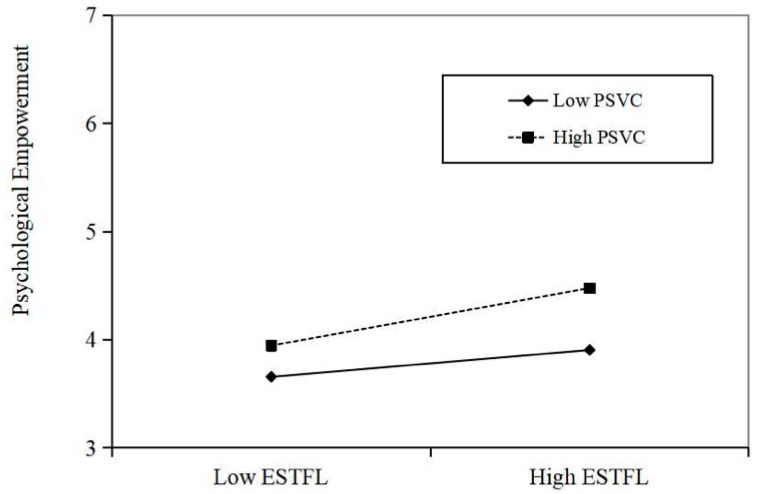
The effect of ESTFL on PE at high and low levels of PSVC.

**Figure 3:**
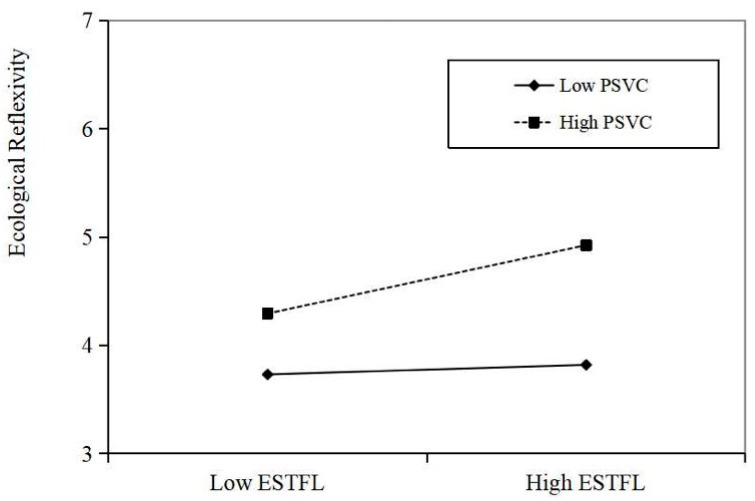
The effect of ESTFL on ER at high and low levels of PSVC.

Hypotheses 3a and 3b indicated the moderation effects of PSVC on the relationship between ESTFL and PE as well as ESTFL and ER. As shown in Table 5, we found significant interactions between ESTFL and PSVC in predicting PE (B = 0.17, *p* < 0.001) and ER (B = 0.32, *p* < 0.001). Meanwhile, we employed the simple slope based on one standard deviation below or above the mean to exhibit the interactions in Figure 2 and Figure 3. As shown in Figure 2, the positive effect of ESTFL on PE was more significant when the PSVC was high. Similarly, Figure 3 demonstrated a more positive relationship between ESTFL and ER among employees with high PSVC. Accordingly, H4a and H4b are supported.

Furthermore, we also tested the moderation effects of PSVC on the indirect effect of ESTFL on GVB via PE and ER. As demonstrated in Table 5, the significant moderated mediation effects with PE (Boot indirect effect = 0.04, Boot SE = 0.02, Boot LLCI = 0.01, Boot ULCI = 0.09) as a mediator and with ER (Boot indirect effect = 0.17, Boot SE = 0.05, Boot LLCI = 0.07, Boot ULCI = 0.26) as a mediator were both verified. Accordingly, Hypotheses 5a and 5b are also supported.

## 5. Discussion

Drawing upon SLT, we developed a moderated dual mediation model to explore how ESTFL influences GVB and when the effect is more effective. Accordingly, these findings further support and extend the current research and theory while also providing effective guidance for leaders and SMEs.

First, the research indicated that ESTFL significantly enhances the employees’ GVB, supporting the results of Crucke et al. (2022). It also supports and expands the argument that transformational leadership is positively linked with green behaviors (Mukhtar et al., 2025). ESTFL, a form of transformational leadership, plays a key role in affecting employee green behaviors (Iqbal et al., 2023; Priatna et al., 2025; Zaid & Yaqub, 2024) including green voice behavior (Crucke et al., 2022). They serve as green role models for their subordinates, and their green attitudes and behaviors will enhance their followers’ environmental consciousness (Othman et al., 2025), thus making their subordinates more positive about generating and sharing green-related ideas. This finding provides empirical support for SLT, which indicates that employees observe and imitate their leaders’ behaviors, ultimately fostering their engagement in environmental voice behavior (Aboramadan et al., 2023).

Second, the study showed that the influence of ESTFL on GVB is partially mediated by dual cognitive mechanisms. Specifically, PE, as an efficacy expectancy, and ER, as an outcome expectancy, both positively mediate the relationship between ESTFL and GVB. Previous research has verified the positive effect of transformational leadership on employee voice behavior through PE (Duan et al., 2017; Ilyas et al., 2021). Moreover, Rietze and Schölmerich (2025) also demonstrated that PE can act as a motivational pathway between green transformational leadership and GVB. Our study is in line with the prior findings and further extends this finding to the green management field. ESTFL leaders can enhance their employees’ sense of autonomy, efficacy, and meaning in work, thereby stimulating them to express ideas and opinions on green matters to others. In addition, ESTFL leaders can also stimulate their employees to reflect upon past work and plan for future actions, thus encouraging them to voice more positively. This aligns with the theoretical assumption of SLT, which points out that leaders often influence employees through two cognitive processes: efficacy expectation and outcome expectation (Bandura, 1977; Wang et al., 2018).

Third, we also found that PSVC moderates the impacts of ESTFL on PE and ER, which demonstrates that when followers and their supervisors share similar green values, ESTFL can enhance PE and ER more effectively. Furthermore, the indirect effects of ESTFL on GVB through PE and ER are also moderated by PSVC. When PSVC is higher, the indirect relationships between ESTFL and GVB via PE and ER are stronger. These findings are in line with previous studies, which posited that PSVC is of great importance in strengthening the effects of leaders’ behaviors on employee outcomes (Elsaied, 2020; He et al., 2024; Lee et al., 2017; Srivastava & Singh, 2023). Based on these prior findings, we further verified the moderated role of PSVC in the relationship between ESTFL on GVB through PE and ER. Moreover, it aligns with SLT, which suggests that the similarity of leaders and followers can significantly influence the efficacy of social learning (Li & Xu, 2019).

### 5.1. Theoretical Implications

This study significantly deepens our understanding of the driving mechanisms behind GVB and extends the literature regarding SLT.

First, it contributes to the research on GVB. In the last decade, studies have established a consensus on the importance and necessity of employees’ green extra-role behaviors (Kim et al., 2017; Zhao et al., 2021). However, they mainly focused on OCBE or other green-related extra-role behaviors (Priyadarshini et al., 2023). Research specifically focusing on GVB remains scarce (S. Hu et al., 2025). According to Van Dyne and LePine (1998), GVB is a challenging and promotive extra-role behavior that is different from other types. It is risky due to its change-oriented nature (Murillo-Ramos et al., 2024), thereby making employees unwilling to express their ideas and opinions. Accordingly, the study offers further insights into the mechanisms of GVB based on SLT. Specifically, ESTFL leaders can serve as inspiring examples to encourage followers to voice green ideas, which reinforces the previous conclusion that ESTFL enhances the employees’ green behaviors (Crucke et al., 2022; Iqbal et al., 2023; Mughal et al., 2022; Priatna et al., 2025). Additionally, employees with high PE and ER will make suggestions more positively because they feel a greater sense of safety, autonomy, and competence.

Second, the present research enriches the mediating mechanisms between ESTFL and GVB based on SLT. It verifies that ESTFL can satisfy the employees’ efficacy expectation through PE, which can help them overcome the fear of voicing (Rietze & Schölmerich, 2025). Meanwhile, it can also improve the employees’ outcome expectation through ER, which makes them believe that their suggestions will matter. They eliminate the two major barriers to voicing behavior, resulting in employees’ transition from “silence” to “voice”. Therefore, the dual mechanisms together provide a more comprehensive perspective for understanding the relationship. Moreover, ER, as a relatively novel concept in the environmental management field (J. Liu et al., 2024), has been largely overlooked despite its potential to deal with some key green-related issues. Thus, this study not only provides a new perspective to understand employee GVB but also further demonstrates the effect of ER in green management.

Third, the study also enriches the boundary mechanism between ESTFL and GVB based on SLT. Previous research has indicated that individual values are crucial for us to understand the relationship between leaders and followers (Brown & Trevino, 2009). Therefore, by testing the moderating role of PSVC, we further reveal the influence of learning contextual differences on the relationship between ESTFL and GVB. When employees share similar values and beliefs with their leaders, they tend to trust their leaders and view them as role models, which means that they will be more confident and reflexive, thereby engaging in GVB.

Fourth, by investigating a dual-cognitive mechanism of PE and ER through which ESTFL impacts GVB, we enrich and deepen the understanding and application of SLT. Previous empirical research mainly explored social learning processes from the perspective of efficacy expectancy, ignoring the outcome expectancy (Li & Xu, 2019). The results reveal that employees working with ESTFL leaders are more likely to engage in GVB because PE can satisfy the employees’ self-efficacy expectation (Guo et al., 2021), while ER can improve their outcome expectancy. This empirically supports one of SLT’s core arguments that the social learning process is realized through two cognitive expectations (Bandura, 1977) and extends its application scope to green management.

### 5.2. Practical Implications

This study mainly focused on SMEs in China. Compared with large enterprises, SMEs may face greater challenges due to resource constraints. Therefore, it is crucial for them to fully leverage the employees’ role in sustainable development. The study offers several managerial insights that are of great practical significance for leaders and Chinese SMEs.

First, the findings imply that ESTFL can promote GVB. Therefore, SMEs should emphasize the importance of ESTFL (Iqbal et al., 2023), especially in China, which has a high power distance culture (Hofstede, 1997). On the one hand, they can identify or set role models who commit to sustainability. For instance, SMEs can select and appoint new leaders with ESTFL characteristics, or they can provide professional training for their current leaders. On the other hand, firms should also praise or reward the contributions of leaders to green management, which further strengthens the benefits of proposing green ideas and taking action. In addition, leaders also need to actively show their green values and behaviors to their followers.

Second, SMEs should promote the employees’ PE level. For example, leaders and firms can encourage positive participation in green affairs and provide necessary and multiple training sessions concerning green knowledge and skills. Furthermore, they should pay attention to the challenges that employees encounter in implementing green initiatives and appreciate their creative ideas related to environmental issues. These can further enhance their self-efficacy to conduct GVB.

Third, SMEs should enhance the employees’ ER level. On the one hand, they can convey the organizations’ green visions, goals, and policies to their employees, which offers enough information for their employees to reflect on current environmental issues. On the other hand, they can regularly hold forums that focus on reviewing and discussing green-related issues to stimulate ER among employees. Furthermore, SMEs should positively adopt constructive ideas proposed by employees and establish incentive mechanisms, thus enhancing their expectations for outcomes. Consequently, employees will be greatly motivated to conduct GVB.

Fourth, the findings indicate that when PSVC is higher, employees can understand the expectations of their leaders better, thus leading to strong GVB. Therefore, organizations should emphasize the value consistency between employees and their leaders. When firms recruit new employees, they should focus on both their abilities and values. Additionally, SMEs can provide training to cultivate green values among their leaders and employees. Furthermore, leaders should communicate their green values and beliefs with their followers positively to strengthen mutual understanding, which may make employees more willing to express their opinions proactively.

Last but not least, SMEs can provide diverse and flexible communication channels for employees, which can create a conducive climate, making employees more comfortable and willing to voice their environmental opinions and suggestions. For example, they can encourage their employees to discuss relevant topics in routine meetings or training sessions, as well as motivate employees to express their ideas via emails, official websites, or social media publicly or anonymously. Certainly, leaders can also encourage their followers to offer their suggestions and ideas on environmental matters face-to-face.

### 5.3. Limitations and Future Research

Despite the study made several contributions to this field, it still had some flaws and limitations. First, all our participants were from SMEs in China, which limited its generalization. Given that China is characterized as a high power distance culture, employees are more likely to be affected by their leaders. Hence, future research needs to be conducted in different cultural contexts. Second, we only employed the questionnaire survey method in this research. Given that filling in questionnaires might be influenced by many unrelated factors, future studies could use multiple methods (e.g., case study and experimental research) to enhance the assessment of causality. Third, the study only examined the mediating role from cognitive dimensions based on SLT. Subsequent studies could investigate the effects of emotional and behavioral mediators such as green harmonious passion, emotional exhaustion, and job satisfaction (Ilyas et al., 2021). Finally, we only explored the contextual moderating effect of PSVC. Other potential boundary mechanisms may be taken into consideration in the future such as organizational climate, proactive personality, and green technology (Liang et al., 2017).

## Figures and Tables

**Table 1:** The demographic descriptions of the participants.

Characteristics	Categories	Employees		Supervisors	
		Frequency	Percentage (%)	Frequency	Percentage (%)
Gender	Male	320	60.40%	73	68.90%
	Female	210	39.60%	33	31.10%
AGE	Below the age of 30	174	32.80%	0	0.00%
	30–40	334	63.00%	52	49.10%
	Above the age of 40	22	4.20%	54	50.90%
Education	High school or below	10	1.90%	0	0.00%
	Junior college degrees	36	6.80%	0	0.00%
	Bachelor’s degrees	470	88.70%	95	89.60%
	Master’s degrees or above	14	2.60%	11	10.40%
Tenure (in years)	1–5	352	66.40%	3	2.80%
	5–10	173	32.60%	87	82.10%
	Above 10	5	1.00%	16	15.10%
Total		530		106	

**Table 2:** The results of confirmatory factor analyses.

Measurement Model	χ^2^	df	χ^2^/df	CFI	TLI	SRMR	RMSEA
Five-factor model + CMV	201.69	120	1.68	0.98	0.97	0.04	0.04
Five-factor model	252.76	125	2.02	0.96	0.95	0.03	0.04
Four-factor model (PE + ER)	480.38	129	3.72	0.89	0.87	0.05	0.07
Four-factor model (ESTFL + PE)	527.20	129	4.09	0.88	0.85	0.06	0.08
Four-factor model (ESTFL + ER)	609.65	129	4.73	0.85	0.82	0.07	0.08
Three-factor model (ESTFL + ER + PE)	767.42	132	5.81	0.80	0.77	0.07	0.10
Two-factor model (ESTFL + ER + PE + PSVC)	940.41	134	7.02	0.75	0.71	0.08	0.11
One-factor model (ESTFL + ER + PE + PSVC + GVB)	965.95	135	7.16	0.74	0.71	0.08	0.11

**Note:** N = 530. **Abbreviations:** CMV, latent common method variance factor; CFI, comparative fit index; TLI, Tucker–Lewis index; RMSEA, root mean square error of approximation; SRMR, standardized root mean square residual; ESTFL, environmentally-specific transformational leadership; PE, psychological empowerment; ER, ecological reflexivity; PSVC, person-supervisor value congruence; GVB, green voice behavior.

**Table 3:** Descriptive statistics, correlations, and validity analysis.

Variables	M	SD	AVE	CR	Correlations								
					1	2	3	4	5	6	7	8	9
1. Gender	1.40	0.49	-	-	-								
2. Age	31.55	3.92	-	-	−0.02	−							
3. Education	2.92	0.40	-	-	0.02	0.15 **	−						
4. Tenure	3.77	1.75	-	-	−0.07	0.71 **	0.12 **	−					
5. ESTFL	3.78	0.50	0.43	0.90	−0.11 *	−0.01	0.11 *	0.03	**0.66**				
6. PE	3.74	0.52	0.41	0.89	−0.03	−0.09 *	0.07	−0.03	0.35 **	**0.64**			
7. ER	3.82	0.78	0.60	0.85	−0.06	−0.04	0.00	−0.01	0.19 **	0.51 **	**0.77**		
8. PSVC	3.76	0.86	0.68	0.86	0.00	0.03	0.09 *	0.04	0.31 **	0.43 **	0.48 **	**0.82**	
9. GVB	3.78	0.87	0.67	0.86	−0.05	0.01	0.13 **	0.08	0.39 **	0.49 **	0.61 **	0.64 **	**0.82**

**Note:** N = 530. * *p* < 0.05, ** *p* < 0.01. The bold numbers on the diagonal represent the square root values of AVE. **Abbreviations:** AVE, average variance extracted; CR, composite reliability; ESTFL, environmentally-specific transformational leadership; PE, psychological empowerment; ER, ecological reflexivity; PSVC, person-supervisor value congruence; GVB, green voice behavior.

**Table 4:** Regression results for the mediation model.

Variables	PE		ER		GVB	
Constant	2.69 ***		3.13 ***		−1.26 **	
Employee gender	0.01		−0.06		0.03	
Employee age	−0.02 *		−0.01		−0.01	
Employee education	0.06		−0.02		0.19 **	
Employee tenure	0.01		0.01		0.05 *	
ESTFL	0.35 ***		0.29 ***		0.39 ***	
PE					0.27 ***	
ER					0.55 ***	
The mediation role of PE/ER on the relationship between ESTFL and GVB						
	Coeff		LLCI		ULCI	
Total effect	0.65		0.51		0.76	
Direct effect	0.39		0.28		0.51	
Indirect effect	PE			ER		
	Coeff	LLCI	ULCI	Coeff	LLCI	ULCI
	0.10	0.04	0.16	0.16	0.04	0.29

**Note:** N = 530. Bootstrap sample size = 5000. * *p* < 0.05; ** *p* < 0.01; *** *p* < 0.001. **Abbreviations:** ESTFL, environmentally-specific transformational leadership; PE, psychological empowerment; ER, ecological reflexivity; GVB, green voice behavior.

**Table 5:** Regression results for the moderation and moderated mediation models.

Predictor	PE				ER			
	B	SE	t	*p*	B	SE	t	*p*
Moderation model								
Constant	4.00 ***	0.22	17.99	<0.001	4.19 ***	0.33	12.63	<0.001
Gender	0.00	0.04	−0.01	>0.05	−0.07	0.06	−1.20	>0.05
Age	−0.02 *	0.01	−2.22	<0.05	−0.01	0.01	−0.85	>0.05
Education	0.06	0.05	1.18	>0.05	−0.02	0.07	−0.31	>0.05
Tenure	0.01	0.02	0.59	>0.05	0.01	0.02	0.25	>0.05
ESTFL	0.39 ***	0.05	8.06	<0.001	0.36 ***	0.07	4.97	<0.001
PSVC	0.25 ***	0.02	10.45	<0.001	0.49 ***	0.04	13.54	<0.001
ESTFL × PSVC	0.17 ***	0.03	5.65	<0.001	0.32 ***	0.04	7.21	<0.001
Moderated mediation model								
PSVC	Indirect effect	Boot SE	Boot LLCI	Boot ULCI	Indirect effect	Boot SE	Boot LLCI	Boot ULCI
Index	0.04	0.02	0.01	0.08	0.17	0.05	0.07	0.26
Conditional indirect effect at PSVC = M ± 1 SD								
M − 1SD	0.07	0.02	0.02	0.11	0.05	0.06	−0.08	0.16
M + 1SD	0.14	0.05	0.05	0.24	0.35	0.10	0.11	0.51

**Note:** N = 530. Bootstrap sample size = 5000. * *p* < 0.05; *** *p* < 0.001. **Abbreviations:** ESTFL, environmentally-specific transformational leadership; PE, psychological empowerment; ER, ecological reflexivity; PSVC, person-supervisor value congruence; GVB, green voice behavior.

## Data Availability

Data will be made available upon reasonable request.
